# Supplementary material for: Modeling glioblastoma heterogeneity as a dynamic network of cell states
Source: Mol Syst Biol. 2021 Sep 16;17(9):e10105. doi: 10.15252/msb.202010105 (PMC8444284; doi:10.15252/msb.202010105)
Supplement: Supplementary file 6 — Source Data for Figure 5 [file MSB-17-e10105-s004.zip › Figure5A_sourcedata/GSEA_3017/hallmarks_stateC.GseaPreranked.1621934520804/HALLMARK_KRAS_SIGNALING_UP.html]

Details for gene set HALLMARK\_KRAS\_SIGNALING\_UP[GSEA]

|  || Dataset | state13017 |
| Phenotype | NoPhenotypeAvailable |
| Upregulated in class | na\_neg |
| GeneSet | HALLMARK\_KRAS\_SIGNALING\_UP |
| Enrichment Score (ES) | -0.4101363 |
| Normalized Enrichment Score (NES) | -1.5742834 |
| Nominal p-value | 0.04707792 |
| FDR q-value | 0.07746749 |
| FWER p-Value | 0.452 |
Table: GSEA Results Summary

  

Fig 1: Enrichment plot: HALLMARK\_KRAS\_SIGNALING\_UP      
 Profile of the Running ES Score & Positions of GeneSet Members on the Rank Ordered List

  

| PROBE | GENE SYMBOL | GENE\_TITLE | RANK IN GENE LIST | RANK METRIC SCORE | RUNNING ES | CORE ENRICHMENT || 1 | GADD45G |  |  | 33 | 0.467 | 0.0151 | No |
| 2 | ETV1 |  |  | 44 | 0.438 | 0.0653 | No |
| 3 | NIN |  |  | 105 | 0.331 | 0.0131 | No |
| 4 | CCND2 |  |  | 149 | 0.288 | -0.0165 | No |
| 5 | APOD |  |  | 222 | -0.259 | -0.1004 | No |
| 6 | PLAU |  |  | 294 | -0.274 | -0.1802 | No |
| 7 | TMEM158 |  |  | 315 | -0.284 | -0.1710 | No |
| 8 | PPP1R15A |  |  | 342 | -0.293 | -0.1706 | No |
| 9 | HBEGF |  |  | 480 | -0.386 | -0.3466 | Yes |
| 10 | CA2 |  |  | 518 | -0.448 | -0.3412 | Yes |
| 11 | IGFBP3 |  |  | 538 | -0.493 | -0.2980 | Yes |
| 12 | PLAT |  |  | 556 | -0.538 | -0.2445 | Yes |
| 13 | SPARCL1 |  |  | 557 | -0.544 | -0.1609 | Yes |
| 14 | SPP1 |  |  | 577 | -0.674 | -0.0898 | Yes |
| 15 | EMP1 |  |  | 589 | -0.785 | 0.0120 | Yes |
Table: GSEA details [plain text format]

  

Fig 2: HALLMARK\_KRAS\_SIGNALING\_UP: Random ES distribution      
 Gene set null distribution of ES for **HALLMARK\_KRAS\_SIGNALING\_UP**
